# Age-dependent effect of metabolic phenotypes on carotid atherosclerotic disease in coronary heart disease patients (CORDIOPREV study)

**DOI:** 10.1186/s12877-020-01544-5

**Published:** 2020-04-22

**Authors:** Francisco M. Gutierrez-Mariscal, Antonio García-Ríos, Purificación Gómez-Luna, Carolina Fernández-Gandara, Magdalena P. Cardelo, Silvia de la Cruz-Ares, Fernando Rodriguez-Cantalejo, Raul M. Luque, Ana León-Acuña, Javier Delgado-Lista, Pablo Perez-Martinez, Elena M. Yubero-Serrano, Jose Lopez-Miranda

**Affiliations:** 1 Lipids and Atherosclerosis Research Unit, Avda. Menéndez Pidal, s/n, Reina Sofia University Hospital, Maimonides Institute for Biomedical Research in Cordoba (IMIBIC), University of Córdoba, 14004 Córdoba, Spain; 2 CIBER Physiopathology of Obesity and Nutrition (CIBEROBN), Carlos III Health Institute, Madrid, Spain; 3 Biochemical Laboratory, Reina Sofia University Hospital, Córdoba, Spain; 4 Department of Cell Biology, Physiology and Immunology, Maimonides Institute for Biomedical Research in Cordoba (IMIBIC), University of Córdoba, Reina Sofia University Hospital, Córdoba, Spain

**Keywords:** Metabolically healthy obese, Cardiovascular risk factors, Metabolic flexibility, Carotid atherosclerosis

## Abstract

**Background:**

Aging is associated with a high risk for cardiovascular disease. The relation of obesity and risk of cardiovascular events appears to be more closely linked to certain clinical or metabolic phenotypes than to obesity itself. Our aim was to establish whether aging influenced the metabolic phenotypes regarding to cardiovascular risk, evaluated by changes in the intima media thickness-common carotid (IMT-CC), in coronary heart disease (CHD) patients.

**Methods:**

In this cross-sectional study, 1002 CHD patients were studied at entry from the CORDIOPREV study. We performed carotid ultrasound assessment to obtain their IMT-CC values. Carotid atherosclerosis was considered to exist if IMT-CC > 0.7 mm.

**Results:**

Age determined a higher IMT-CC, regardless metabolic phenotype (all *p* < 0.05). Metabolically healthy non-obese (MHNO) aged< 60 showed a lesser prevalence for carotid atherosclerotic disease than metabolically sick non-obese (MSNO) and obese (MSO), while MHNO aged≥60 only showed less prevalence for the disease than the MSO. Carotid atherosclerosis associated with age, sex, impaired fasting glucose (IFG), hypertension and high sensitivity C-reactive protein (hsCRP). However, in patients aged< 60, it associated with sex and IFG and in the age ≥ 60 group, with hypertension and hsCRP.

**Conclusions:**

Our results suggest that CHD patients aged≥60 are less metabolic flexible compared to patients aged< 60. Thus, MHO patients aged≥60 show the same risk of suffering carotid atherosclerosis as those with metabolic disease, while MHO patients aged< 60 show lower risk than MSO. This fact indicates the need to focus on therapeutic strategies in order to modify those parameters related to obesity and metabolic inflexibility in patients with CHD before entering old age.

## Background

Aging can be defined as a natural process in which the chances of survival of an organism gradually decline. It is a multifactorial process involving genetic and environmental factors [22, 30]. Aging is associated with an increased risk of suffering age-related diseases such as diabetes, cancer, neurodegenerative diseases or dementia, and is also widely considered as a cardiovascular risk factor [25]. It is common knowledge that body weight tends to increase with age, except in the elderly, while the prevalence of metabolic disease is higher among older individuals, particularly those with obesity [3, 17].

Obesity, defined as abnormal or excessive body fat accumulation, is related to an increased risk of cardiometabolic diseases [21, 31]. Over the past decades, it has increased worldwide as is now considered a pandemic, not only in the middle-aged or pediatric population, but also in the elderly [7, 29]. There is increasing evidence to suggest that not all individuals in obese populations display metabolic disorders and a high cardiovascular risk associated with obesity and, conversely, not all normal-weight individuals are in a favorable metabolic condition [10]. In fact, our current knowledge suggests that it is not obesity itself, but certain clinical or metabolic phenotypes associated with it, which are linked to an increase of the cardiovascular risk [12, 15, 28]. In this context, the so-called metabolically healthy obese phenotype is characterized by increased body fat and a favorable cardiovascular profile (high levels of HDL-cholesterol, normal glucose and triglyceride levels and good insulin sensitivity) [11, 26]. However, normal-weight individuals with metabolic disease show early signs of insulin resistance, hyperinsulinemia, atherogenic dyslipidemia and hypertension, with a high susceptibility to develop diabetes and cardiovascular disease (CVD) [11, 15]. However, to date, it is not known whether the associated cardiovascular risk with metabolic phenotypes could be modified by age.

Taking all the above into account, the aim of the present study was to establish whether age influences the cardiovascular risk associated with metabolic phenotypes (the presence/or absence of obesity and/or metabolic disease), measured by intima-media thickness of both the common carotid arteries (IMT-CC), as marker of carotid atherosclerosis, in a large cohort of coronary heart disease (CHD) patients. The identification of these potential associations would provide evidence to allow the use of more intensive medical interventions and prevention strategies aimed at reducing CVD risk and delay an age-related decline in health.

## Methods

### Population

This work has been carried out in the setting of the CORDIOPREV study (Clinical Trials Registry NCT00924937). The study protocol was designed in accordance with the principles of the Declaration of Helsinki, approved by the Human Investigation Review Committee of the Reina Sofia University Hospital, and according to institutional and Good Clinical Practice guidelines. The CORDIOPREV study is described in depth in previously published report [6]. For the specific aim of this work, we performed a cross-sectional analysis of the CORDIOPREV population at baseline and the patients were classified according to: 1) Age, up and under the median of our studied population, 60 years; 2) the presence/or absence of obesity [body mass index (BMI) < 30 and ≥ 30] and 3) metabolic disease (described below). From the initial sample of 1002 subjects, we included only 939 subjects: those whose carotid ultrasound, analytical and anthropometric data were available. The reasons for the lack of data for the remaining 63 patients were as follows: 37 refused to undergo the echography, 14 withdrew from the study before conducting the test and 12 for other reasons [28].

### Metabolic phenotypes

The different metabolic phenotypes included in the study were defined as follows: 1. Metabolically healthy non-obese (MHNO): BMI < 30 and < 2 factors of cardiometabolic criteria. 2. Metabolically healthy obese (MHO): BMI ≥ 30 and < 2 factors of cardiometabolic criteria. 3. Metabolically sick non-obese (MSNO): BMI < 30 and ≥ 2 factors of cardiometabolic criteria. 4. Metabolically sick obese (MSO): BMI ≥ 30 and ≥ 2 factors of cardiometabolic criteria. Description of cardiometabolic disease criteria are in supplemental material.

### Laboratory tests

At 8.00 am, following a 12-h fast, the patients were admitted to the laboratory for anthropometric and biochemical tests [BMI, waist circumference, Waist to Height Ratio (WHTR), systolic blood pressure (SBP), diastolic blood pressure (DBP), HDL-cholesterol, LDL-cholesterol, triglycerides, cholesterol, high sensitive C-reactive protein (hsCRP), glucose, hemoglobin A1c (HbA1c) and homeostatic model assessment for insulin resistance (HOMA-IR)]as described in supplemental material.

### Intima media thickness assessment

All the patients were examined in supine position with the neck hyper-extended and the chin turned to one side. The carotid arteries were examined bilaterally using a Doppler ultrasound high-resolution B-mode (Envisor C Ultrasound System, Phillips, USA), following the recommendations of the American Society of Echocardiography Carotid Intima-Media Thickness Task Force [27]. The observers were unaware of the patients’ demographic and cardiovascular risk data during the assessment. The measurements were taken using semi-automatic software (QLAB Advance Ultrasound Quantification Software, v5.0, Phillips, USA). Three measurements were taken for each patient, and we obtained the general mean IMT-CC. Carotid atherosclerotic disease was defined by IMT-CC ≥0.7 mm [18, 23]. This cutoff value was used to calculate the prevalence of carotid atherosclerotic disease as a percentage of the patients with IMT-CC ≥0.7 mm from the total number of patients in each group analyzed.

### Statistical analysis

The statistical analyses were carried out using SPSS version 19.0 for Windows (SPSS Inc., Chicago, IL, USA). Continuous data were compared by analysis of variance (ANOVA) in which smoking habit and sex were included as covariates. Categorical variables were compared using Chi Square tests. In order to assess the association between the phenotypes and the presence of carotid atherosclerotic disease, an odds ratios (OR) analysis was performed. Furthermore, backward multiple lineal and logistic analysis were carried out to estimate the association between cardiovascular risk factors and IMT-CC values. The differences were considered significant when *p* < 0.05. All the data presented in figures and tables are expressed as means ± standard error (SE).

## Results

### Study population characteristics based on age and metabolic phenotypes

In the analysis of the influence of age and metabolic phenotypes on biochemical and anthropometrics characteristics of the population (Shown in Supplemental Table 1), waist circumference and cholesterol showed a differential effect (*p <* 0.05): MSO patients aged< 60 years showed the highest waist circumference and MSNO and MSO patients aged≥60 years exhibited the lowest cholesterol. Furthermore, MSO patients aged≥60 years had higher insulin and HbA1c levels, but fewer BMI compared to those aged< 60 years (*p* < 0.05); MHO patients aged≥60 years showed higher HDL-cholesterol levels compared to those aged< 60 years (*p* < 0.05). In both age groups, MSO patients showed higher BMI, HOMA-IR, HbA1c and hsCRP levels compared to the rest of metabolic phenotypes (all *p* < 0.05). However, only MSO patients aged< 60 years showed higher waist circumference, glucose and insulin levels, and lower HDL-cholesterol levels, compared to the rest of metabolic phenotypes (all *p <* 0.05). In the analysis of WHTR, a biomarker of fat visceral deposit, age > 60 years determined higher value than age < 60 years, being all of them equal or upper 0.6 points which shows high risk of fat visceral localization with a subsequent loss in metabolic flexibility. In each group of age, we observed that in those age > 60 years obesity determined higher WHTR than non-obese, however in age < 60 years the highest value for this parameter belongs to those MSO.

### Differences in IMT-CC between metabolic phenotypes are influenced by age

**Fig. 1:**
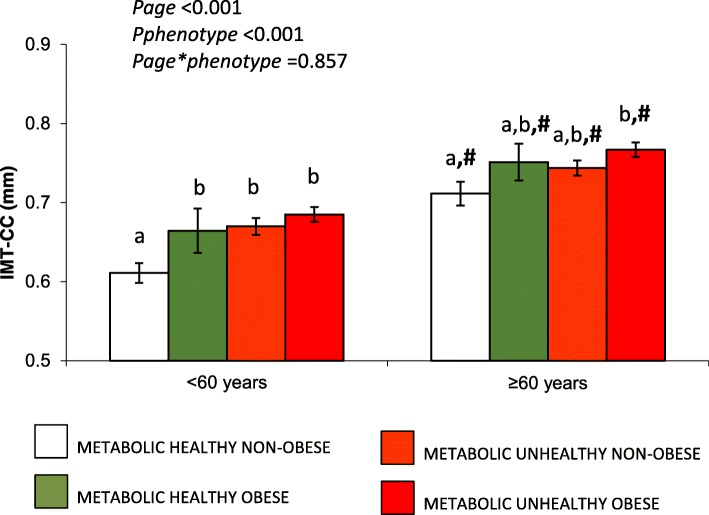
Influence of metabolic phenotypes and age on IMT-CC. All data are mean +/− SE. *p* < .05, Continuous variables were compared using the analysis of variance (ANOVA). #*p* < .05 aged< 60 years vs aged≥60 years. Bars with different letters a and b mean differences between metabolic phenotypes. IMT-CC: Intima media thickness-common carotid

Regarding to the influence of metabolic phenotypes on IMT-CC, age resulted in a higher IMT-CC regardless of the metabolic phenotype (all *p* < 0.05) (Fig. 1). In the group of patients aged< 60 years, MHNO had the lowest IMT-CC compared to the other phenotypes (p < 0.05). In the group of patients aged≥60 years, MHNO showed the lowest IMT-CC, while MSO patients presented the highest IMT-CC (*p <* 0.05), with intermediate values for IMT-CC in MHO and MSNO patients (Fig. 1).

### Age produces differences in the prevalence of carotid atherosclerotic disease according to metabolic phenotypes

In order to find a link between the different metabolic phenotypes and the presence or not of carotid atherosclerotic disease, defined as IMT-CC ≥0.7 mm, we calculated OR for both the whole study population and according to age. Table 1 shows the prevalence of carotid atherosclerotic disease and OR values. When we evaluated the whole population, the MHNO patients presented the lowest prevalence of carotid atherosclerotic disease compared to the rest of phenotypes. In patients aged< 60 years, MHNO showed less prevalence of carotid atherosclerosis than MSNO and MSO phenotypes, but not MHO. However, in patients aged≥60 years, MHNO showed less prevalence of carotid atherosclerosis than MSO, but not less than the other phenotypes (Table 1).

**Table 1:** Prevalence of carotid atherosclerosis diseases according to metabolic phenotypes and its association study by Odds Ratios

	MHNO	MHO	MSNO	MSO	χ^**2**^ Test
**All population (*n*** **= 939)**	**109**	**71**	**305**	**454**	***p*** **= 0.003**
**Carotid Atherosclerosis disease prevalence (IMT-CC ≥ 0.7 mm)**	33.9%^a^	49.3%^b^	49.2%^b^	54.0%^b^	
**Odds Ratio**	1	**1.89 (1.03–3.49)**	**1.88 (1.19–2.97)**	**2.28 (1.47–3.53)**	
**Age < 60 (*n*** **= 435)**	**58**	**26**	**141**	**210**	***p*** **= 0.028**
**Carotid Atherosclerosis disease prevalence (IMT-CC ≥ 0.7 mm)**	19.0%^a^	30.8%^a, b^	36.2%^b^	40.0%^b^	
**Odds Ratio**	1	1.90 (0.66–5.48)	**2.42 (1.15–5.08)**	**2.85 (1.40–5.81)**	
**Age ≥ 60 (*n*** **= 504)**	**51**	**45**	**164**	**244**	*p* = 0.209
**Carotid Atherosclerosis disease prevalence (IMT-CC ≥ 0.7 mm)**	51.0%^a^	60.0%^a, b^	60.4%^a, b^	66.0%^b^	
**Odds Ratio**	1	1.44 (0.64–3.24)	1.46 (0.78–2.75)	**1.87 (1.02–3.42)**	

Carotid Atherosclerosis disease (carotid intima media thickness ≥ 0.7 mm) prevalence is expressed in percentage. Odds ratio values are calculated using MHNO phenotype as reference (95% confidence interval). Odds Ratio in bold mean that all values in the confidence interval are higher than 1. χ^2^ Test performed to compare prevalence of carotid atherosclerosis disease between metabolic phenotypes. Percentages in a row with superscripts with different letters a and b mean differences between phenotype group

Regarding to the OR analysis, we observed that in the whole population, MHO, MSNO and MSO phenotypes were associated with the presence of carotid atherosclerosis, taking the MHNO phenotype as a reference. In patients aged< 60 years, the MHO phenotype was not associated with a higher prevalence of the disease compared to MHNO (OR < 1), but this association did appear with the presence of metabolic disease (OR = 2.42 for MSNO and 2.85 for MSO), although the value of prevalence of carotid atherosclerosis disease was seen to increase from MHNO to MSO. However, in the group of patients aged≥60 years, MHO and MSNO did not result in a higher probability of the disease than MHNO, while MSO showed an association with the presence of the disease [OR: 1.87 (1.02–3.42)].

### Multiple regression analysis

In order to study which biochemical and anthropometric parameters s are involved in carotid atherosclerotic disease among the different metabolic phenotypes, we performed a multiple logistic regression analysis (Table 2). In this regard, the presence of carotid atherosclerosis in the whole population seems to be associated with age, sex, IFG, hypertension and hsCRP. However, when we looked for these associations in each group split by age, we observed that in patients aged<60 years, carotid atherosclerosis was associated with sex and IFG, while in patients aged≥60 years, it appeared to be associated with hypertension and hsCRP.

**Table 2:** Multiple logistic regression

Variables	Coefficients	SE	p	Odds Ratio	95% CI
**The whole population**					
**Age**	**1.118**	**0.157**	**0.000**	**3.059**	**2.248–4.162**
**Sex**	**0.868**	**0.219**	**0.000**	**2.382**	**1.550–3.662**
**IFG**	**0.455**	**0.155**	**0.003**	**1.577**	**1.163–2.138**
hsCRP	0.303	0.166	0.067	1.359	0.979–1.874
**Hypertension**	**0.477**	**0.163**	**0.003**	**1.611**	**1.170–2.218**
WHTR	1.897	1.135	0.095	6.666	0.720–61.672
**Patients Age < 60 years**					
**Sex**	**0.849**	**0.414**	**0.041**	**2.337**	**1.037–5.264**
**IFG**	**0.836**	**0.228**	**0.000**	**2.308**	**1.478–3.605**
**Patients Age ≥ 60 years**					
**Sex**	**0.880**	**0.263**	**0.001**	**2.411**	**1.440–4.038**
**Hypertension**	**0.831**	**0.225**	**0.000**	**2.295**	**1.476–3.571**
**hsCRP**	**0.478**	**0.231**	**0.039**	**1.612**	**1.024–2.537**
WHTR	2.830	1.526	0.064	16.940	0.850–337.420

*R*^*2*^ = 0.167, constant = −3.251 (*p* = 0.000). Alcohol consumption, smoking, LowHDL-c, HOMA-IR, HyperTG, Obesity and Waist Circumference have been eliminated from the model (*p* > 0.05)

*R*^*2*^ = 0.072, constant = −1.859 (*p* = 0.000). Alcohol consumption, smoking, Obesity, LowHDL-c, HOMA-IR, HyperTG, Waist Circumference, Hypertension and hsCRP have been eliminated from the model (*p* > 0.05)

*R*^*2*^ = 0.099, constant = −2.785 (*p* = 0.008). Alcohol consumption, smoking, Obesity, LowHDL-c, HOMA-IR, HyperTG, IFG and Waist Circumference have been eliminated from the model (*p* > 0.05)

Multiple Logistic Regression with Age, Sex, IFG, LowHDL-c, hsCRP, HOMA-IR, Hypertension, Obesity, Waist Circumference, *WHTR* Waist to Height Ratio, *HyperTG* Alcohol consumption and Smoking as variable proved in the model. *IFG* Impaired Fasting Glucose, *hsCRP* high sensitive C-reactive protein, *HOMA-IR* homeostatic model assessment for insulin resistance, *HyperTG* Hypertrigliceridemia

## Discussion

This study provides new findings about risk factors for carotid atherosclerosis associated with metabolic status and obesity, defined by metabolic phenotypes, and its relationship with age in patients with CHD. Our data highlights the age-dependency of this association and suggests that the relative importance of metabolic parameters and obesity differ in patients below and over 60 years old. Therefore, in patients aged< 60 years, there is an increased risk of carotid atherosclerosis among the MSO, MSNO and MHO groups compared to MHNO, where IFG seems to be the most influential metabolic parameter. However, in patients aged≥60 years, those classified as MSO showed the highest risk for the disease, being hypertension, hsCRP and WHTR (a biomarker of fat visceral deposit) [24] the metabolic parameters that more contributed to the risk for carotid atherosclerosis. In the study of predictive and earlier biomarkers for CVD, IMT-CC is considered a surrogate marker that allows the anatomical changes in the arterial wall to be explored and quantified [14, 16] and can be used as an indicator of the presence of carotid atherosclerosis [5, 13]. Among risk factors studied regarding the development of carotid atherosclerotic disease, weight is considered an independent risk factor for CVD and is associated with higher mortality [1]. Obesity, and particularly central obesity, increases the risk of CVD and is associated with diabetes, hypertension and dyslipidemia, as well as other lifestyle risk factors such as physical inactivity and poor diet [20]. In this area, Choi et al. found that older MSNO adults showed a markedly higher risk of all-cause mortality, whereas overweight or obese subjects without metabolic disease had a comparatively lower risk of death [4].

The MHO phenotype has been described as an intermediate stage between a healthy and sick status, producing the same risk of suffering a cardiovascular event as MHNO [2]. Although this has been noted in most published studies, in the present study we observed a higher association with carotid atherosclerosis for the MHO phenotype than for MHNO. However, when we analyzed patients according to age, MHO patients aged< 60 years showed no link to a higher prevalence of carotid atherosclerosis. Moreover, the MSNO and MSO phenotypes showed a higher prevalence than MHNO. Indeed, an increasing prevalence of carotid atherosclerosis can be seen between MHNO and MSO.

Recent data from a 20-year follow up suggest that cumulative incidence rates for CHD, stroke and survival probability in individuals with suboptimal health (≤two metabolic diseases factors) were intermediate between the healthy and unhealthy subgroups, which was not affected by the BMI [8]. In contrast to our results, accordance exists in the whole population and in patients aged< 60 years, but not aged≥60 years, suggesting that, with the progression of age, there may be a loss in metabolic flexibility, where the presence of obesity could trigger, a loss in the capacity for lipogenesis with an increase of visceral fat depots coming from a dysfunctional adipose tissue, transforming the metabolically healthy obese in a phenotype associated with a higher risk for carotid atherosclerosis. In fact, the measure of fat visceral depots, through the WHTR, depicts how those age ≥ 60 years had more than 0.6 which determines a high risk of fat visceral contributing to the development of metabolic inflexibility.

Hamer M et al. reported, in a stability study regarding the healthy obese phenotype, that unstable healthy obese (those who develop unhealthy status at the following 8 years) showed greater increases in central adiposity and impaired glycaemic control with slightly elevated levels of HbA1c [9]. In agreement with this study, in the multiple logistic regression analysis, we found that the impairment of glycaemic control, such as IFG, was associated with the presence of the disease, but only in patients aged<60 years. However, we showed that in those aged≥60 years, the association appeared for hypertension, increased hsCRP and WHTR. Here, therefore, our results showed up the evident different importance of these parameters in the high risk for carotid atherosclerosis observed among the metabolic phenotypes according to age. In a previous study from our group in this population, we demonstrated how certain types of the metabolic phenotypes are less favorable modulating phenotypic flexibility [19]. This fact suggests us that one of the mechanisms behind these effects, described above, could be related to the loss of capacity for lipogenesis with increasing adipose tissue and the gaining weight that occurs in adulthood. In those aged<60, these moderate changes in weight could be mitigated by maintaining these capacities which do not increase the numbers of risk factors. Surprisingly, smoking status (non-smoker, current smoker and ex-smoker) is kept out from all the models. Despite the fact that smoking is considered as an influential variable which is widely described in the literature, we must take into account the characteristics of our population, who are patients with CHD who have suffered a cardiovascular event, of which the percentage of active smokers is extremely low (< 10%) since they have all been previously recommended to give up smoking.

One of the limitations found in our study is that it is cross-sectional, which offers no evidence of causal effects, but instead provides a link between the diseases and the variables studied. On the other hand, this analysis could provide the basis for future approaches in longitudinal studies in order to clarify the influence of metabolic phenotypes in the development of carotid atherosclerosis and CVD measured by IMT-CC. In this context,

it is therefore important to identify the potential risk factors associated to the CVD risk and monitor them in the change from adulthood to old age. According to that, our study is focused on the analysis of this association, by assessing atherosclerotic disease, which is behind and preceding the development of cardiovascular events, so it could provide practitioners with information about their patients to act preventing the event or even mortality. It seems clear that high cardiovascular risk groups, such as MHO and, especially, those age ≥ 60 years, would benefit from risk factor stratification to identify obese individuals who may gain most from modifying specific metabolic parameters, with the subsequent benefits for their metabolic health profile and a reduction in development of cardiometabolic disease.

## Conclusion

In conclusion, our results suggest that in CHD patients, there is an age-dependent effect of metabolic phenotypes on carotid atherosclerotic disease. CHD patients aged≥60 years lose their metabolic flexibility compared to patients aged< 60 years. This fact indicates the need to focus on therapeutic strategies in order to modify those parameters related to obesity and metabolic inflexibility in patients with CHD before entering old age.

## Supplementary information

**Additional file 1.**

## Data Availability

The datasets used and/or analyzed during the current study are available from the corresponding author on reasonable request.

## Abbreviations

ANOVA: Analysis of Variance
BMI: Body Mass Index
CHD: Coronary Heart Disease
CVD: Cardiovascular Disease
DBP: Diastolic Blood Pressure
HbA1c: Hemoglobin A1c
HOMA-IR: Homeostatic Model Assessment for Insulin Resistance
hsCRP: High sensivity C-Reactive Protein
IFG: Impaired Fasting Glucose
IMT-CC: Intima Media Thickness-Common Carotid
MHNO: Metabolically Healthy non-Obese
MHO: Metabolically Healthy Obese
MSNO: Metabolically Sick non-Obese
MSO: Metabolically Sick Obese
O: Odds Ratio
SBP: Systolic Blood Pressure
SE: Standard Error
WHTR: Waist to Height Ratio
