# Supplementary material for: Age-dependent effect of metabolic phenotypes on carotid atherosclerotic disease in coronary heart disease patients (CORDIOPREV study)
Source: BMC Geriatr. 2020 Apr 22;20:151. doi: 10.1186/s12877-020-01544-5 (PMC7178935; doi:10.1186/s12877-020-01544-5)
Supplement: Supplementary file 1 — Additional file 1. [file 12877_2020_1544_MOESM1_ESM.docx]

**Title: Age-dependent effect of metabolic phenotypes on carotid atherosclerotic disease in coronary heart disease patients (CORDIOPREV study)**

Francisco M. Gutierrez-Mariscal PhD^1,2^, Antonio García Ríos MD, PhD^1,2^, Purificación Gómez Luna PhD^1,2^, Carolina Fernández-Gandara BS^1,2^, Magdalena P Cardelo BS^1,2^, Silvia de la Cruz-Ares PhD^1,2^, Fernando Rodriguez-Cantalejo MD^3^, Raul M. Luque PhD^2,4^, Ana León-Acuña MD, PhD^1,2^, Javier Delgado-Lista MD, PhD^1,2^, Pablo Perez-Martinez MD, PhD^1,2^, Elena M. Yubero-Serrano PhD^1,2*^, Jose Lopez-Miranda MD, PhD^1,2*^

^1^Lipids and Atherosclerosis Unit, Maimonides Institute for Biomedical Research in Cordoba, Reina Sofia University Hospital, University of Córdoba, Córdoba, Spain.

^2^CIBER Physiopathology of Obesity and Nutrition (CIBEROBN), Carlos III Health Institute, Madrid, Spain.

^3^Biochemical Laboratory, Reina Sofia University Hospital, Córdoba, Spain

^4^Department of Cell Biology, Physiology and Immunology, Maimonides Institute for Biomedical Research in Cordoba, University of Córdoba, Reina Sofia University Hospital, Córdoba, Spain.

**Address for correspondence: Jose Lopez-Miranda**. Reina Sofia University Hospital, IMIBIC/Lipids and Atherosclerosis Research Unit. Avda. Menéndez Pidal, s/n. 14004 Córdoba, Spain. Phone: +34-957010947. FAX: +34-957218250. e-mail: [jlopezmir@uco.es](mailto:jlopezmir@uco.es).

**SUPPLEMENTAL MATERIAL**

**METHODS**

**Laboratory tests**

At 8.00 am, following a 12-h fast, the patients were admitted to the laboratory for anthropometric and biochemical tests [BMI, waist circumference, Waist to Height Ratio (WHTR), Systolic blood pressure (SBP), Diastolic blood pressure (DBP), HDL-cholesterol, LDL-cholesterol, triglycerides, cholesterol, high sensitive C-reactive protein (hsCRP), glucose, hemoglobin A1c (HbA1c) and homeostatic model assessment for insulin resistance (HOMA-IR)]. The patients had refrained from smoking during the fasting period and abstained from alcohol intake for the past 7 days. Venous blood was sampled from the antecubital vein, collected in Vacutainer tubes with no anticoagulant and into tubes containing EDTA, and tubes were immediately transferred to 4º C. To minimize proteolytic degradation, the plasma was supplemented with protease inhibitor cocktail (Roche Diagnostic, Germany) 40 µL per mL of plasma. The plasma and serum samples were frozen at -80º C for further biochemical analysis. The serum parameters were measured in Architect c-16000 analyzers (Abbott®, Chicago, IL, USA) using spectrophotometric techniques (enzymatic colorimetric methods): hexokinase method for glucose, and oxidation–peroxidation for cholesterol; the triglycerides, LDL-cholesterol and HDL-cholesterol levels were estimated using the Friedewald formula based on cholesterol, triglycerides, and HDL-cholesterol concentrations. The plasma levels of insulin were measured by chemiluminescent microparticle immunoassay using an analyzer (i-2000 Abbott Architect®, Chicago, IL, USA). The plasma concentrations of hsCRP were determined by high sensitivity ELISA (BioCheck, Inc., Foster City, CA, USA). HOMA-IR was derived from fasting insulin (µU/L) x fasting glucose (µmoles/L)/22.5.

|  | Age < 60 years | | | | Age ≥ 60 years | | | | |  | |
| --- | --- | --- | --- | --- | --- | --- | --- | --- | --- | --- | --- |
|  | MHNO  (n = 58) | MHO  (n = 26) | MSNO  (n = 141) | MSO  (n = 210) | MHNO  (n = 51) | MHO  (n = 45) | MSNO  (n = 164) | MSO  (n = 244) | P(age*phenotypes) | |  |
| Age (years) | 50.6 ± 0.8 | 51.1 ± 1.2 | 51.8 ± 0.5 | 51.6 ± 0.4 | 67.3 ± 0.7^*^ | 67.0 ± 0.8^*^ | 65.9 ± 0.3^*^ | 66.4 ± 0.3^*^ | 0.130 | |  |
| BMI (kg/m^2^) | 26.5 ± 0.3^a^ | 32.7 ± 0.5^b^ | 27.5 ± 0.2^a^ | 34.8 ± 0.3^c^ | 26.8 ± 0.3^a^ | 32.5 ± 0.3^b^ | 27.5 ± 0.2^a^ | 33.9 ± 0.2^*,c^ | 0.115 | |  |
| Waist circumference (cm) | 93.3 ± 1.0^a^ | 105.3 ± 1.6^b^ | 96.9 ± 0.7^a^ | 112.1 ± 0.8^c^ | 98.8 ± 1.3^*,a^ | 108.8 ± 1.2^b^ | 98.3 ± 0.7^*,a^ | 111.9 ± 0.6^b^ | 0.031 | |  |
| SBP (mmHg) | 124 ± 2.2^a^ | 123 ± 2.8^a^ | 135 ± 1.4^b^ | 136 ± 1.3^b^ | 133 ± 2.6^*,a^ | 131 ± 2.5^a^ | 144 ± 1.5^*,b^ | 147 ± 1.4^*,b^ | 0.818 | |  |
| DBP (mmHg) | 75 ± 1.3^a^ | 75 ± 1.6^a,b^ | 81 ± 0.9^b^ | 80 ± 0.8^b^ | 73 ± 1.5^a^ | 69 ± 1.5^*,a^ | 76 ± 0.8^*,a,b^ | 77 ± 0.7^*,a,b^ | 0.659 | |  |
| LDL-C (mg/dL) | 88.1 ± 3.2 | 87.6 ± 4.1 | 92.1 ± 2.4 | 88.8 ± 1.8 | 91.1 ± 3.9 | 93.7 ± 3.5 | 86.2 ± 2.0^*^ | 84.4 ± 1.5^*^ | 0.115 | |  |
| HDL-C (mg/dL) | 47.5 ± 1.5^a^ | 44.6 ± 1.5^a,b^ | 41.6 ± 0.8^b^ | 38.7 ± 0.6^c^ | 50.7 ± 1.3^a^ | 50.4 ± 1.5^*,a^ | 41.9 ± 0.8^b^ | 40.2 ± 0.6^b^ | 0.058 | |  |
| TG (mmol/L) | 88.1 ± 3.4^a^ | 88.7 ± 5.1^a^ | 150.6 ± 7.5^b^ | 167.1 ± 5.9^b^ | 88.5 ± 3.9^a^ | 92.0 ± 3.9^a^ | 132.2 ± 5.6^*,b^ | 140.9 ± 4.2^*,b^ | 0.179 | |  |
| Cholesterol (mg/dL) | 155.1 ± 4.0 | 150.3 ± 4.7 | 165.7 ± 3.1 | 160.3 ± 2.0 | 161.2 ± 4.2 | 162.9 ± 4.4 | 154.4 ± 2.6^*^ | 154.4 ± 1.8^*^ | 0.003 | |  |
| Glucose (mg/dL) | 87.4 ± 1.3^a^ | 94.0 ± 2.8^a,b^ | 109.6 ± 3.2^b^ | 123.2 ± 3.3^c^ | 91.5 ± 1.7^a^ | 89.4 ± 1.1^a^ | 116.2 ± 2.5^b^ | 125.0 ± 2.8^b^ | 0.701 | |  |
| Insulin (mU/L) | 4.7 ± 0.3^a^ | 8.5 ± 0.8^a,b^ | 9.2 ± 0.6^b^ | 15.4 ± 1.0^c^ | 5.6 ± 0.5^a^ | 6.4 ± 0.5^a^ | 9.1 ± 0.7^a^ | 12.6 ± 0.9^*,b^ | 0.191 | |  |
| HbA1c (%) | 5.9 ± 0.1^a^ | 6.1 ± 0.1^a,b^ | 6.5 ± 0.1^b,c^ | 6.8 ± 0.1^c^ | 6.2 ± 0.1^a^ | 6.2 ± 0.1^a,b^ | 6.7 ± 0.1^b^ | 7.0 ± 0.1^*,c^ | 0.983 | |  |
| hsCRP (mg/L) | 1.6 ± 0.4^a^ | 1.5 ± 0.2^a,b^ | 3.3 ± 0.4^b,c^ | 3.9 ± 0.3^c^ | 1.5 ± 0.3^a^ | 1.9 ± 0.2^a,b^ | 2.8 ± 0.3^a,c^ | 3.4 ± 0.2^b,c^ | 0.750 | |  |
| HOMA-IR | 1.0 ± 0.1^a^ | 1.9 ± 0.2^a,b^ | 2.6 ± 0.2^b^ | 4.1 ± 0.2^c^ | 1.3 ± 0.1^a^ | 1.4 ± 0.1^a^ | 2.8 ± 0.2^b^ | 3.8 ± 0.2^c^ | 0.510 | |  |
| WHTR | 0.56 ± 0.01^a^ | 0.64 ± 0.01^b^ | 0.58 ± 0.01^a^ | 0.67 ± 0.01^c^ | 0.60 ± 0.01^*,a^ | 0.67 ± 0.01^*,b^ | 0.60 ± 0.01^*,a^ | 0.69 ± 0.01^*,b^ | 0.319 | |  |

**Supplemental Table 1. Biochemical and anthropometrics characteristics of the population according to age and metabolic phenotypes.**

Values represent means ± SE. Continuous variables were compared using the analysis of variance (ANOVA). Qualitative variables were compared using Chi Square test. MHNO: Metabolically healthy non-obese; MHO: Metabolically healthy obese; MSNO: Metabolically sick non-obese; MSO: Metabolically sick obese; BMI: Body mass index; SBP, Systolic blood pressure; DBP, Diastolic blood pressure; LDL-C, low density lipoprotein-cholesterol; HDL-C, high-density lipoprotein-cholesterol; TG, triglycerides; HbA1c, hemoglobin A1c; hsCRP, high sensitive C-reactive protein; HOMA-IR: homeostatic model assessment for insulin resistance; WHTR: Waist-to-Height Ratio. ^*^*p* < 0.05 Age≥60 years *vs* Age ˂60 years (ANOVA for repeated measures). Means in a row with superscripts without a common letter differ significantly within an age group.
